# Impact of donor graft quality on deep anterior lamellar Keratoplasty (DALK)

**DOI:** 10.1186/s12886-017-0600-6

**Published:** 2017-11-17

**Authors:** Friederike Schaub, Philip Enders, Werner Adler, Björn O. Bachmann, Claus Cursiefen, Ludwig M. Heindl

**Affiliations:** 1 Department of Ophthalmology, University of Cologne, Kerpener Strasse 62, 50924 Cologne, Germany; 2 Department of Medical Informatics, Biometry and Epidemiology, Friedrich-Alexander University Erlangen-Nürnberg, Waldstr. 6, 91054 Erlangen, Germany

**Keywords:** DALK, Donor tissue, Eye bank, Keratoconus, Split cornea transplantation

## Abstract

**Background:**

To evaluate main features of donor tissue that may influence clinical outcome or complication rate after deep anterior lamellar keratoplasty (DALK).

**Methods:**

Donor tissue parameters of 84 consecutive corneal donor grafts used for big-bubble DALK surgery between June 2011 and December 2014 in 84 eyes of 84 patients with disorders of anterior corneal stroma were correlated to clinical outcome parameters of recipient eyes 12 months after surgery and 3 months after total suture removal. Main donor tissue parameters included age), post-mortem time, overall preservation time, preservation time after split and prior to transplantation, and preservation technique. Clinical outcome parameters included best spectacle corrected visual acuity (BSCVA), central corneal thickness (CCT), endothelial cell density (ECD) and complication rates. Pearson’s correlation, linear regression analysis for clinical outcome parameter and logistic regression analysis for postsurgical complication rates were applied.

**Results:**

Corneal donors were mean aged 67.4 ± 12.5 years with a post-mortem time of 20.7 ± 14.7 h and ECD of 2641.0 ± 362.8 cells/mm^2^. Overall preservation time was 16.3 ± 6.3 days. Recipients showed mean BSCVA 12 months postoperatively of 0.60 ± 0.36 logMAR, endothelial cell loss was 4 ± 16%, and central corneal thickness was 571.7 ± 54.2 μm. 3 months after total removal of sutures, BSCVA was 0.20 ± 0.10 logMAR, endothelial cell loss was 17 ± 24%, and central corneal thickness was 590.9 ± 55.5 μm. Loosening of sutures occurred in 20%, and Descemet detachment in 16%. None of the clinical outcome parameters or complication rate after DALK showed a significant association with donor tissue parameters.

**Conclusions:**

Donor corneas, independent of excision techniques or preservation method, with donor age ≤ 88 years, post-mortem time ≤ 63 h, overall preservation time ≤ 14 days for cold storaged donor tissue and ≤35 days for organ culture, and preservation time after split prior to grafting ≤96 h, seem to be applicable as safe donor tissue for DALK surgery.

## Background

Since the introduction of different lamellar keratoplasty techniques the claims to the “perfect” donor graft vary between different indications and methods of corneal transplantation.

Traditionally, penetrating keratoplasty (PK) as full-thickness replacement of the cornea has been the standard technique for corneal transplantation in advanced stages of keratoconus, anterior stromal opacity or endothelial failure. Consequently, donor tissue had to be clear without stromal haze, epithelial or endothelial pathologies [1–3].

In 1997 deep anterior lamellar keratoplasty (DALK) was introduced, and there has been a steady increase in its uptake during the last years [4–6]. Nowadays, DALK seems to be the superior approach for anterior corneal pathology, especially in keratoconus, in eyes with a healthy endothelium as it eliminates the possibility of endothelial rejection that may result in graft failure [3, 6, 7].

Comparative analyses between DALK and PK for keratoconus report varying results. Graft survival and outcome seem to be affected by several factors, including host and donor factors, improvement in surgical technique, and possibly surgeons experience and learning curve [6, 8, 9].

Most patients with keratoconus are young and therefore have a higher demand on their visual function. Therefore, it is of particular importance to further optimize results of DALK surgery, respectively to find out what can be assigned as important basic requirements of donor tissue.

Furthermore, eye banks have to consider carefully about the distribution of donor tissue to potential recipients with various corneal pathologies, especially because of the enormous donor shortage in many parts of the world, including Europe and Asia [10–13]. Accordingly, it would be of great interest to precisely define “quality features” for donor grafts for the different methods of keratoplasty, especially for lamellar corneal transplantation techniques.

The aim of this study was to evaluate retrospectively whether donor tissue features, including donor tissue excision techniques, preservation methods, preservation time and previously performed stripping of the donor button for split cornea transplantation for combined use in Descemet membrane endothelial keratoplasty (DMEK) and DALK may influence the clinical outcome and complication rate of DALK surgery.

## Methods

In this retrospective study, we reviewed the database of the local eye bank of the Department of Ophthalmology, University of Cologne, Cologne, Germany for all donor grafts, that were assessed and distributed for DALK surgery, as well as the corresponding clinical records of 84 eyes of 84 patients who underwent DALK surgery at the Department of Ophthalmology, University of Cologne, Cologne, Germany, between June 2011 and December 2014. The retrospective, nonrandomized, clinical study was approved by the institutional review board (15–301) and was conducted in adherence to the tenets of the Declaration of Helsinki.

### Collection of donor graft data

Prior to donor graft distribution and transplantation the following data were assessed: age and gender of donor, cause of death, central corneal thickness (μm), endothelial cell density (cells/mm^2^), post-mortem time (hours), donor tissue excision technique (corneoscleral disc excision technique versus whole globe enucleation technique), preservation technique (organ culture versus 4 °C preservation) and preservation time until grafting (days). If the donor graft was used for split cornea transplantation, the preservation time prior to split (days) and between split and prior to transplantation (hours) was assessed separately.

Central corneal thickness was measured by ultrasound pachymetry (Quantel Medical SAS, Pocket-II; Cournon d’Auvergne Cedex, France), and endothelial cell density by phase-contrast microscopy (Axiovert 25, Zeiss; Oberkochen, Germany).

### Clinical information and collected data of recipients

Prior to surgery, 12 months postoperatively and 3 months after total removal of corneal sutures, standardized eye examinations, including best spectacle-corrected visual acuity (BSCVA), manifest subjective refraction (trial glasses in a trial frame), topographic astigmatism (SimK’s measured with Orbscan, Bausch & Lomb), central corneal thickness (Orbscan, Bausch & Lomb), endothelial cell density (Tomey EM-3000 Specular Microscope), slit lamp examination and funduscopy were performed. Intraoperative and postoperative complications were recorded.

### Inclusion and exclusion criteria

Exclusion criteria of the local eye bank for cornea donation were: Age ≥ 90 years, as well as post-mortem time ≥ 72 h and positive infectious status in serology or in the anterior chamber probe. Assessment of infectious status in serology was performed in a blood sample which has been taken inbetween 24 h post mortem or in a sample which was still present from the lifetime. Prior to approval for transplantation the following information had to be completed and checked: negative status of serologic screening tests for transmissible diseases, evaluation of donated cornea, commitment to tissue donation by next relatives or the presence of a written commitment of the deceased, and corneal procurement following medical standards.

The surgeons performing DALK had no further specific demands on the donor lenticule, beside transparency of the graft without stromal scars. If cornea split transplantation, combining DALK and DMEK, was intended, donor tissue was supposed to have at best endothelial cell density ≥ 2000 cells/mm^2^.

Cases in which DALK was intended but conversion to PK was needed due to intraoperative macroperforation were excluded. Further exclusion criteria were history of any keratoplasty in the past, known history of severe ocular trauma, recurrent keratitis of unknown origin, uveitis, significant immunosuppression, steven-johnson syndrome or graft-versus-host-disease.

If both eyes in one patient underwent DALK surgery, one eye and respective donor cornea was randomly selected for the study.

All donor corneas used for big-bubble DALK surgery meeting in- and exclusion criteria were included consecutively in the present.

For statistical analysis concerning BSCVA, partial exclusion criteria were amblyopia or other visual acuity limiting factors such as existence of macular degeneration or advanced stage of glaucoma. 74 eyes could be considered for analysis of BSCVA, 10 eyes were excluded.

### Distribution of donor grafts, donor preparation and surgical technique

Donor tissues were allocated by the eye bank staff. Main factors of distribution of donor grafts to recipients were the availability of donor tissue and urgency of corneal transplantation.

All patients underwent surgery under full anesthesia and under steady state conditions in the eye hospital. DALK was performed in a standardized fashion using the big-bubble technique as described previously [14, 15]. In cases where no big-bubble could be achieved, baring of the Descemet membrane (DM) was achieved by dissecting the stroma in layers using the “microbubble incision technique” [4, 6, 16].

If possible, split cornea transplantation was performed. In split cornea transplantation the anterior part of the donor button was used for DALK, the posterior part for DMEK in another patient [10, 12, 17, 18]. Depending on the availability of the patients, either DALK or DMEK was performed first. The remaining donor lenticule was grafted within one week.

The Descemet membrane (DM) was peeled off with fine nontoothed forceps. The donor lenticule was secured with 10–0 monofilament nylon by 16 interrupted sutures or a 16-bite double-running diagonal cross-stitch suture.

### Postoperative course

All patients received routine postoperative management in the form of topical prednisolone acetate 1% in tapering doses over several months and topical antibiotic drops for 1 to 2 weeks (in most cases moxifloxacin). The steroid medication tapered to two times a day after 3 months and discontinued after 1 year. Half of the sutures were removed 12 months after DALK, the remaining sutures after 18 months. Often the time point of suture removal was adapted to factors as wound healing and vascularization, loose sutures or astigmatism.

### Statistical analyses

Descriptive data for donor tissue and recipients were collected and analyzed by Microsoft Excel 2000 for windows. For further statistical analysis, we used SPSS (version 22.0 for windows; SPSS, Inc., Chicago, IL). The BSCVA results were analyzed in logarithm of the minimum angle of resolution (logMAR) equivalent units. The following statistical tests were applied: exact Fisher test, Mann-Whitney U test, and Pearson’s correlation. The level of significance was defined as *p* < 0.05, and adjusted according to the Benjamini-Hochberg correction of multiple testing.

Linear and logistic regression analysis were performed using the using the programming language RV 3.2.2 (R Foundation for Statistical Computing, Vienna, Austria).

## Results

### Donor details

Donors had a mean ± SD age of 67.4 ± 12.5 years (range, 17–88 years), and a female to male ratio of 28:56. Mean ± SD post-mortem time was 20.7 ± 14.7 h (range, 4–63 h). Eleven (13%) donor corneas were harvested by corneoscleral disc excision and 73 donor corneas (87%) by whole globe enucleation. Eleven donor corneas (13%) were stored at 2–8 °C in Optisol GS (Bausch & Lomb Surgical, Inc., Rochester, NY, USA) or Life 4 °C (Chiron Vision, Irvine, CA, USA), and 73 donor corneas (87%) were organ cultured (Biochrome, Berlin, Germany) at 31 ± 1 °C.

Mean ± SD overall preservation time was 16.3 ± 6.3 days (range, 6–31 days). Hereby donor corneas stored in Optisol GS or Life 4 °C had a mean ± SD overall preservation time of 11 ± 1.7 days (range, 9–14 days) and organ cultured donor corneas of 17.3 ± 6.4 days (range, 8–35 days). Mean ± SD preservation time after split and prior to transplantation was 6.0 ± 16.8 h (range, 0–96 h), whereas mean ± SD preservation time after split and prior to transplantation was 1.7 ± 1.4 h (range, 0–4 h) for cold storaged donor tissue and 6.6 ± 18.0 h (range, 0–96 h) for organ cultured tissue. Mean ± SD endothelial cell density of donor lenticule was counted as 2641.0 ± 362.8 cells/mm^2^ (range, 1022–3500 cells/mm^2^), mean ± SD central corneal thickness was measured as 617.7 ± 6.3 μm (range, 470–915 μm).

In 67 eyes (80%) split cornea transplantation could be performed, thereof 9 donor buttons were cold storaged (13%) and 58 were organ cultured (87%). In 9 cases (13%) of split cornea transplantation DALK was performed first so that donor tissues were stripped intraoperatively directly prior to DALK procedure. In 58 (87%) eyes pre-stripped donor tissues were used.

### Clinical outcome

In 84 eyes of 84 patients (female to male ratio: 19:65; mean age ± SD 42.4 ± 15.9 years (range, 18–78 years)) DALK was performed. Indications included advanced keratoconus in 67 eyes, corneal scarring in 13 eyes, and lattice corneal dystrophy in 4 eyes. Secondary diagnosis included history of glaucoma (*n* = 1), previously performed cataract surgery (*n* = 7), amblyopia (*n* = 9), mild trauma (*n* = 3), keratoconjunctivitis sicca (*n* = 2), history of herpetic keratitis (*n* = 10) and status after laser-assisted in situ keratomileusis (LASIK) (*n* = 1) or phototherapeutic keratectomy (PTK) (n = 1).

76 eyes had follow-up evaluations for 12 months follow-up; only data of 8 eyes were not available. In 44 eyes total removal of sutures was completed, out of those 42 eyes were amenable for the second postoperative evaluation (3 months after total removal of sutures).

Mean ± SD best spectacle corrected visual acuity was 0.91 ± 0.47 logMAR (range, 0.1–2.0 logMAR) prior to corneal transplantation, 0.60 ± 0.36 logMAR (range, 0.1–2.0 logMAR) at 1 year follow-up, and 0.20 ± 0.10 logMAR (range, −0.10 - 0.30 logMAR) 3 months after suture removal.

Changes in mean ± SD spherical equivalent were −3.3 ± 6.3 dpt prior to DALK to −2.6 ± 4.1 dpt after total removal of all sutures. Mean ± SD topographic astigmatism (SimK’s measured with Orbscan, Bausch & Lomb) changed from −6.7 ± 4.0 dpt to −3.45 ± 2.1 dpt, respectively.

Mean ± SD central corneal thickness was 442.9 ± 130.1 μm (range, 183–1112 μm) prior to corneal transplantation, 571.7 ± 54.2 μm (range, 434–792 μm) at 1 year follow-up, and 590.9 ± 55.5 μm (range, 450–675 μm) at final investigation.

Endothelial cell density preoperatively was 2408 ± 392.1 cells/mm^2^, whereas 12 months postoperatively 2264 ± 350 cells/mm^2^, and 3 months after suture removal 2040 ± 462 cells/mm^2^. Mean ± SD endothelial cell loss 12 months after DALK was 4 ± 16%, and 3 months after total suture removal 17 ± 24%.

### Complications

Seventeen eyes (20%) showed loosened sutures during the observation period, and in 13 eyes (16%) Descemet detachment occurred due to intraoperative microperforation of the DM. A poor ocular surface leading to persistent epithelial defect (lasting >2 weeks despite treatment) was seen in 7 eyes (8%). Five of 7 responded to medical treatment and/or bandage contact lens. In 2 of 7 eyes amniotic membrane transplantation and tarsorrhaphy were necessary in cases of ulcerative keratitis. Response to these treatments was seen in 1 eye, but in the other eye only initial response could be observed, subsequently recurrent epithelial defects developed. During the course there was corneal perforation, which required PK à chaud. There was no case of primary graft failure or graft rejection, respectively. Further postoperative complications in recipient eyes are shown in Table 1.

**Table 1:** Descriptive data of postsurgical complications

Complications	Total number of eyes	%
Loosening of sutures	17	20%
- early (first 6 months after surgery)	10	12%
- middle (7–12 months after surgery)	5	6%
- late (> 12 months after surgery)	2	2%
Descemet detachment	13	16%
- early re-bubbling (day 1–7)	8	62%
- late re-bubbling (> day 7)	4	31%
- conservatively managed	1	8%
Persistent Descemet folds	12	14%
Persistent epithelial defect (> 14 days)	7	8%
Suture-related microbial keratitis	3	4%
Wound leakage requiring resuturing	3	4%
Interface haze	3	4%
Interface vascularisation	3	4%
Graft infiltrate	2	2%
Primary graft failure	0	0%
Graft rejection	0	0%

For each complication the number of affected eyes and corresponding percentages are listed. Complications are listed in descending incidence

### Association of outcome and complications with donor tissue features

None of the clinical outcomes or complication rate after DALK showed a significant association with donor tissue features, including age, gender, post-mortem time, excision technique, preservation time, preservation method, endothelial cell density and central corneal thickness (*p* ≥ 0.338; Table 2).

**Table 2:** Association of complication rate and clinical outcome parameters with donor tissue features

	Donor tissue								
Outcome parameters	Gender	Age	ECD	Pm time	Excision technique	Preservation method	Total preservation time	Preservation time after split	Split
DM Detachment	0.857*	0.857‡	0.974‡	0.338‡	0.857*	0.857*	0.857‡	0.857‡	0.857*
DM folds	0.857*	0.857‡	0.857‡	0.987‡	0.857*	0.857*	0.857‡	0.857‡	0.857*
Persistent epithelial defect	0.857*	0.857‡	0.857‡	0.857‡	0.857*	0.857*	0.857‡	0.943‡	0.857*
Loose sutures	0.857*	0.857‡	0.857‡	0.857‡	0.857*	0.857*	0.857‡	0.857‡	0.857*
BSCVA after 12 months	0.857‡	0.857+	0.857+	0.857+	0.891‡	0.891‡	0.857+	0.857+	0.857‡
BSCVA after suture removal	0.913‡	0.891+	0.974+	0.857+	0.974‡	0.974‡	0.974+	0.857+	0.857‡
EC loss after 12 months	0.857‡	0.857+	0.857+	0.857+	0.857‡	0.857‡	0. 338+	0.857+	0.857‡
EC loss after suture removal	0.857‡	0.857+	0.857+	0.338+	0.857‡	0.857‡	0.857+	0.857+	0.857‡
Central corneal thickness after 12 months	0.338‡	0.857+	0.857+	0.949+	0.857‡	0.857‡	0.857+	0.857+	0.857‡
Central corneal thickness after suture removal	0.857‡	0.857+	0.857+	0.857+	0.974‡	0.974‡	0.857+	0.974+	0.857‡

*P*-values adjusted according to Benjamini-Hochberg correction of multiple testing are listed

* exact Fisher test; ‡ Mann-Whitney U test; + Pearson’s correlation

*DM*, Descemet membrane, *BSCVA*, best spectacle corrected visual acuity, *EC loss* endothelial cell loss, *ECD* endothelial cell density, *Pm time* post-mortem time

Furthermore, in linear regression analyses for the main clinical outcome parameters including BSCVA and CCT (Table 3), as well as logistic regression analyses for most frequent postsurgical complications (Table 4) no association to donor tissue parameters could be revealed in comparison to the donor tissue parameters (*p* ≥ 0.072), respectively.

**Table 3:** Linear regression analysis of clinical outcome parameters

	Donor tissue	Coefficient	95% Confidence interval (CI)	*P*-value
BSCVA after 12 months	Age	0.007	−0.01 – 0.024	0.408
	ECD	0.000	−0.001 – 0.001	0.760
	Total preservation time	−0.008	−0.041 – 0.024	0.604
	Preservation time after split	−0.006	−0.023 – 0.011	0.509
BSCVA after suture removal	age	−0.002	−0.005 – 0.002	0.275
	ECD	0.000	0–0	0.571
	Total preservation time	0.002	−0.005 – 0.009	0.579
	Preservation time after split	0.000	−0.003 – 0.003	0.908
CCT after 12 months	age	−0.489	−1.711 – 0.733	0.426
	ECD	−0.018	−0.061 – 0.025	0.411
	Total preservation time	−1.248	−3.877 – 1.381	0.345
	Preservation time after split	−0.172	−1.45 – 1.106	0.788
CCT after suture removal	age	−0.500	−2.094 – 1.094	0.523
	ECD	−0.023	−0.082 – 0.036	0.422
	Total preservation time	0.921	−3.848 – 5.691	0.693
	Preservation time after split	0.739	−3.164 – 4.641	0.699

Linear regression analysis for main clinical outcome parameters including BSCVA and CCT results of recipients. Independent donor tissue variables: Age, ECD, total preservation time and preservation time after split

*BSCVA* best spectacle corrected visual acuity, *CCT*, central corneal thickness, *CI* Confidence interval, *ECD* endothelial cell density

**Table 4:** Logistic regression analysis for postsurgical complications

	Donor tissue	Odds ratio (OR)	95% Confidence interval (CI)	*P*-value
Loosening of sutures	Age	0.961	0.917–1.007	0.088
	ECD	0.999	0.998–1.001	0.466
	Total preservation time	0.941	0.847–1.047	0.256
	Preservation time after split	0.978	0.898–1.064	0.597
Descemet detachment	age	1.023	0.965–1.086	0.438
	ECD	1.000	0.998–1.002	0.855
	Total preservation time	1.043	0.951–1.143	0.366
	Preservation time after split	0.997	0.947–1.05	0.915
Persistent Descemet folds	age	1.018	0.96–1.081	0.539
	ECD	1.000	0.998–1.002	0.819
	Total preservation time	1.059	0.964–1.163	0.223
	Preservation time after split	0.930	0.752–1.15	0.495
Persistent epithelial defects	age	0.975	0.909–1.046	0.468
	ECD	0.997	0.994–1	0.072
	Total preservation time	0.797	0.622–1.021	0.068
	Preservation time after split	0.946	0.73–1.226	0.670

Logistic regression analysis for most frequent postsurgical complications. Independent donor tissue variables include age, ECD, total preservation time and preservation time after split

*ECD* endothelial cell density, *CI* Confidence interval, *OR* Odds ratio

## Discussion

This study revealed no correlation between quality of donor tissue and clinical outcome in deep anterior lamellar keratoplasty. Donor corneas with donor age ≤ 88 years, post-mortem time ≤ 63 h, endothelial cell densities of ≥1000 cells/mm^2^, overall preservation time of ≤14 days for cold storaged donor tissue or ≤35 days in cases of organ culture, and preservation time after split prior to grafting ≤96 h seem to be applicable as safe donor tissue for DALK surgery. Donor tissue quality harvested by corneoscleral disc excision technique is comparable to donor tissue quality after whole globe enucleation. Organ culture method for preservation is suitable as well as 4 °C storage techniques of donor tissues used for DALK. These results may indicate advantages and particularly more feasibility in distribution of donor tissue for DALK recipients for eye banks and in clinical routine.

The selective replacement of the diseased anatomic structure of the cornea under preservation of the healthy tissue in DALK offers distinct advantages over PK. Deep anterior lamellar keratoplasty shows a reduced risk of endothelial cell rejection resulting from the preservation of the recipient endothelium and a reduced rate of intraocular complications [1, 3, 6]. DALK provides a greater availability of donor corneas that do not need a high density of endothelial cells to be suitable for PK [5, 19].

Our clinical results indicate a good visual outcome with low complication rates, confirming DALK as safe alternative to PK, independent of donor tissue variables.

In particular, as new finding, we can outline, that there is no association between preservation method and clinical outcome after DALK surgery and between donor tissue excision technique and outcome parameters. Observations of Feizi et al. [20] showed, that the use of donor tissue with a lower quality can be used for DALK surgery and can provide good visual outcome. All included donor tissues were preserved at 4 °C with a mean preservation time of 2 days and were stripped directly before DALK surgery. None of those were previously splitted prior to grafting for cornea split transplantation [20]. Up to now, there was no further comparable and detailed investigation of organ cultured donor tissue regarding the evaluation of possible association between donor tissue variables and outcome parameters.

In the literature one can find hints, that a longer post-mortem time and longer storage times may be associated with epithelial defects in the early postoperative period after keratoplasty [20–22]. In our study, regarding epitheliopathies in the context of postoperative complications, we focused on persisting epithelial defects that occurred in 8%. Five of 7 affected eyes responded to medical treatment or bandage contact lens. In statistical analyses no significant association between donor tissue quality and the occurrence of postoperative epithelial defects could be detected.

Early or late loosening of sutures was observed in 17 eyes (20%) and Descemet detachment in 13 eyes (16%). These complication rates appear to be higher than expected. It should be noted, however, that loosening of sutures occur more frequently when interrupted sutures are performed compared to double-running diagonal cross-stitch sutures.

Suture loosening was documented as soon as positive Seidel probe of the stitch channel was present or even in case a suture was removed without a retrospectively recognizable reason. Therefore, the rate could be overestimated. As Descemet detachment, any distance of the Descemet membrane was evaluated, even if this was not clinically significant, i.e. even if no stromal edema resulted. To the best of our knowledge, comparative data regarding these complications are rare, therefore a prospective evaluation would be desirable. However, these complications were without relation to clinical outcome parameters. Altogether, none of the outcome parameters revealed statistical significance.

Although PK for keratoconus has the highest survival of all indications for corneal transplantation, its main drawback is the risk of endothelial rejection. DALK minimizes the risk of endothelial graft rejection, but patients who received DALK remain susceptible to stromal subepithelial types of graft rejections. The frequency of occurrence differs between rates of 3–21% after DALK surgery [3, 6, 23, 24]. There was no case of primary graft failure or graft rejection after DALK in our study. Certainly the sample size may be too small to state that there is no significant connection with the quality of donor tissue regarding this respect.

Although some postoperative complications were observed in a few eyes, no graft was lost due to lower tissue quality. Of course, frequent follow-up examinations and appropriate interventions in complicated cases played an important role.

Borderie et al. examined 166 patients after anterior lamellar keratoplasty at different time points. None of the main donor characteristics, such as endothelial cell density or preservation time, were correlated to clinical outcome. The only factor that showed influence on outcome parameter was donor age. Donor age > 80 years was associated with lower BSCVA. Borderie et al. recommended to exclude donor grafts of older donors for anterior lamellar keratoplasty [19]. In contrast, in our study no significant correlation between visual outcome and donor variables could be found. Our results are consistent with those of Heindl et al. [17] and Feizi et al. [20].

In addition, there was no effect on clinical outcome of preservation time after split and prior to transplantation. Preservation times of the anterior donor lenticule after splitting of up to 96 h were accomplished. Our data are in line with Heindl et al., showing, that splitted donor tissue may be stored safely for up to 1 week in organ culture before use in DALK or DMEK surgery [17]. Also Borderie et al. could not draw any connection between preservation time or deswelling time to any outcome parameters [19]. This fact may open up the possibility to perform more cornea transplantations as split transplantation, even if scheduling of recipients is challenging [10, 12, 25].

The present study revealed that clinical outcome after DALK surgery is independent of the quality of donor tissue and preservation method. It is based on retrospective collected data, which has both advantages and limitations. One limitation is the heterogeneity of recipient eyes including different corneal pathologies of the anterior corneal stroma. Since DALK surgery is performed less frequently than posterior lamellar keratoplasty in our department, inclusion of a larger more homogeneous cohort of eyes seems to be challenging. Furthermore, clinical outcome parameters i.e. BSCVA results are not entirely dependent on the underlying indication for transplantation but the more on comorbidities. Nevertheless, it would be desirable to additionally examine the impact of donor tissue quality in a more homogeneous cohort including additional assessment of the influence of epithelial status and stromal swelling of the donor grafts at the time point of surgery as well as the parameters that have already been investigated retrospectively in a prospective clinical study to define our presumptions more clearly.

## Conclusions

In summary our results support expanding inclusion criteria for potential DALK donor corneas. Especially, for DALK older donors up to 88 years, or longer post-mortem times up to 63 h, independent of tissue excision techniques or preservation methods, should be considered as acceptable in the future to counter donor shortage. Whenever it is possible, split cornea transplantation combining DALK and DMEK should be performed, even if, due to logistical problems, a preservation time of the anterior lenticule after split until grafting is needed.

## Abbreviations

BSCVA: Best spectacle corrected visual acuity
CI: Confidence interval
DALK: Deep anterior lamellar keratoplasty
DM: Descemet membrane
DMEK: Descemet membrane endothelial keratoplasty
EC: Endothelial cells
ECD: Endothelial cell density
LASIK: Laser-assisted in situ keratomileusis
logMAR: Logarithm of the minimum angle of resolution
OR: Odds ratio
PK: Penetrating keratoplasty
Pm time: Post-mortem time
PTK: Phototherapeutic keratectomy
SD: Standard deviation
